# Functional Interaction Between the Oncogenic Kinase NEK2 and Sam68 Promotes a Splicing Program Involved in Migration and Invasion in Triple-Negative Breast Cancer

**DOI:** 10.3389/fonc.2022.880654

**Published:** 2022-04-21

**Authors:** Chiara Naro, Federica Barbagallo, Cinzia Caggiano, Monica De Musso, Valentina Panzeri, Silvia Di Agostino, Maria Paola Paronetto, Claudio Sette

**Affiliations:** ^1^ Department of Neuroscience, Section of Human Anatomy, University of the Sacred Hearth, Rome, Italy; ^2^ Gemelli SCIENCE and TECHNOLOGY PARK (GSTeP)-Organoids Research Core Facility, Fondazione Policlinico Agostino Gemelli IRCCS, Rome, Italy; ^3^ Department of Experimental Medicine, University of Rome Sapienza, Rome, Italy; ^4^ Department of Health Sciences, “Magna Graecia” University of Catanzaro, Catanzaro, Italy; ^5^ Department of Movement, Human and Health Sciences, University of Rome Foro Italico, Rome, Italy; ^6^ Laboratory of Molecular and Cellular Neurobiology, Fondazione Santa Lucia IRCCS, Rome, Italy

**Keywords:** triple-negative breast cancer, alternative splicing, transcriptomics, NEK2, SAM68

## Abstract

Triple-negative breast cancer (TNBC) represents the most aggressive breast cancer subtype. Poor prognosis in TNBC is partly due to lack of efficacious targeted therapy and high propensity to metastasize. Dysregulation of alternative splicing has recently emerged as a trait of TNBC, suggesting that unveiling the molecular mechanisms underlying its regulation could uncover new druggable cancer vulnerabilities. The oncogenic kinase NEK2 is significantly upregulated in TNBC and contributes to shaping their unique splicing profile. Herein, we found that NEK2 interacts with the RNA binding protein Sam68 in TNBC cells and that NEK2-mediated phosphorylation of Sam68 enhances its splicing activity. Genome-wide transcriptome analyses identified the splicing targets of Sam68 in TNBC cells and revealed a common set of exons that are co-regulated by NEK2. Functional annotation of splicing-regulated genes highlighted cell migration and spreading as biological processes regulated by Sam68. Accordingly, Sam68 depletion reduces TNBC cell migration and invasion, and these effects are potentiated by the concomitant inhibition of NEK2 activity. Our findings indicate that Sam68 and NEK2 functionally cooperate in the regulation of a splicing program that sustains the pro-metastatic features of TNBC cells.

## Introduction

Alternative splicing is the molecular process that generates multiple mRNA variants from single eukaryotic genes through variable assortment of their exons (1, 2). This process amplifies the coding potential of genomes and represents a plastic device for the regulation of gene expression. However, errors in alternative splicing regulation are implicated in the pathogenesis of various human diseases, including cancer (3, 4). Integration of transcriptomic analyses with clinical data have documented that genome-wide alterations in splicing occur in many human cancers, as well as the utility of splice variants as diagnostic or prognostic biomarkers (5–7). In this regard, splicing signatures were shown to distinguish breast cancers (BCs) from normal tissue (7) and to clearly segregate the more aggressive triple-negative BC subtype (TNBC) from the other BCs (8–10).

Oncogenic splicing dysregulation mainly relies on the aberrant expression of specific splicing factors (2, 11). For instance, the oncogenic transcription factor MYC directly induces transcription of several genes encoding for splicing factors (12, 13), including *SRSF1* (14). Importantly, overexpression of the SRSF1 protein was sufficient to induce transformation of mammary epithelial cells and such oncogenic activity was shown to rely, at least in part, on the promotion of splice variants that enhance cell survival, proliferation, and migration (15). Another important layer of splicing regulation relies on the control of the activity of splicing factors through their reversible phosphorylation (16, 17). Splicing-specific kinases, such as the serine arginine protein kinase (SRPK) and the CDC-like kinase (CLK) families, and cell-signaling kinases were reported to regulate both splicing factor expression and activity (16, 17). For instance, phosphorylation by the SRPK1, AURKA, and NEK2 kinases was shown to enhance the splicing activity of SRSF1 in multiple cancer cell types (18–20). Thus, deregulated expression of protein kinases represents another important source of pro-oncogenic splicing alterations.

NEK2 is a mitotic kinase that is frequently upregulated in human cancers, where it contributes to malignancy and drug resistance (21–23). Accordingly, high NEK2 expression was correlated with rapid relapse and poor outcome in multiple cancers (21), including BC (24). In this regard, NEK2 is particularly upregulated in TNBC (25), the BC subtype displaying the poorest prognosis due to high metastatic rate and lack of targeted therapies (26, 27). Although NEK2 oncogenic activity in cancer has been primarily associated to the promotion of genome instability and aneuploidy (22, 28–30), mounting evidence suggests its implication in the pro-tumoral regulation of alternative splicing. Indeed, NEK2 was shown to accumulate in the nucleus of cancer cells (18, 21, 31) and to modulate the activity of splicing factors (18, 32). We recently reported that NEK2 localizes in the nucleus of TNBC cells and exerts a widespread impact on the TNBC-specific transcriptome (25). Part of the splicing changes elicited by NEK2 were mediated by regulation of the expression of the splicing factor RBFOX2, which drives a pro-mesenchymal splicing program in TNBC cells (25). However, a substantial fraction of NEK2-regulated exons and introns were devoid of RBFOX2 binding motifs, suggesting that additional molecular mechanisms contribute to NEK2-mediated splicing regulation in TNBC.

In this study, by searching for additional mediators of NEK2-dependent splicing regulation, we found that NEK2 interacts with and phosphorylates the multifunctional RNA binding protein (RBP) Sam68, thus modulating its splicing activity. Transcriptome analysis of TNBC cells transiently silenced for Sam68 identified the splicing targets of this protein in TNBC cells and revealed a common set of exons that are co-regulated by NEK2, which are enriched in genes related to cell migration. Sam68 depletion in TNBC cells reduces migration and matrix invasion and these effects are enhanced by concomitant inhibition of NEK2 kinase activity. Thus, our study suggests that Sam68 and NEK2 functionally cooperate in the regulation of a splicing program that sustains pro-metastatic features of TNBC cells.

## Materials and Methods

### Cell Culture, Treatment, and Transfection

MDA-MB-231 cells were grown in RPMI 1640 (Lonza), SUM159 cells were grown in DMEM/F12 (Sigma Aldrich), and HEK293T cells were grown in DMEM, all supplemented with 10% FBS, gentamycin, penicillin, and streptomycin. Plasmid transfection was performed using Lipofectamine 2000 (Invitrogen) according to the manufacturer’s instruction. For RNA interference, cells were transfected with siRNAs (Sigma-Aldrich) using Lipofectamine RNAiMAX (Invitrogen) according to the manufacturer’s instructions and harvested 48 h later for protein and RNA analyses. Sequences of siRNAs are listed in Additional File 1: **Supplementary Table 1**. c-MYC targeting siRNAs were previously described (13).

### Plasmid Vectors

The expression vector pcDNA3N_2_Myc-NEK2C WT was a generous gift of Prof A. Fry; a catalytically inactive mutant of NEK2C KD (NEK2_K37R_) was created by site-directed mutagenesis of pCDNA3N_2_myc-NEK2C WT. Mutagenic oligonucleotides were as follows: forward, 5’AGATATTAGTTTGGAGAGAACTTGACTATGGC3’; and reverse, with the underlined codon corresponding to residue 37 in wild-type NEK2C. Construction of the pcDNA3N2Myc-NEK2A WT and the kinase-dead inactive mutant NEK2A KD plasmids was previously described (33). The sequence of wild-type and mutant NEK2 were confirmed by sequence analysis. The expression vectors pEGFP NEK2C WT or KD were sub-cloned from pcDNA3N_2_Myc into pEGFPc1. pGEX-3X–NEK2_273–444_ encoding the regulatory domain of NEK2 fused to glutathione *S*-transferase (GST) has been described previously (34). CD44 minigene was a kind gift of Prof. Matter.

### RNA Extraction, Library Preparation, and RNA-Seq Data Analysis

For RNA-seq analysis, MDA-MB-231 transiently transfected with control (si-CTRL) or SAM68-targeting (si-SAM68) pool of siRNA were harvested 48 h after transfection in triplicate and total RNA was extracted and DNase treated using the RNEasy mini kit (Qiagen) according to the manufacturer’s instruction. PolyA plus RNA-seq libraries were constructed according to Illumina’s protocol and sequenced using a 100-bp single-end format on an Illumina HiSeq 2000. RNA-Seq data analysis was performed by GenoSplice technology (www.genosplice.com), as previously described (25, 35), using Human FAST DB v2016_1 annotations. Results were considered statistically significant for *p*-values ≤0.05 and fold-changes ≥1.5.

### Bioinformatic Analysis

Analysis of gene expression of transcriptomic data of TNBC patients from the “The Cancer Genome Atlas (TCGA)” database was performed using the UCSC Xena platform (36). Spearman’s correlation was used to evaluate association between the expression of *NEK2*, *MYC*, and indicated splicing factors. For gene expression analyses, the patients were divided into two groups according to the first (*NEK2*-low) and fourth (*NEK2*-high) quartile of *NEK2* gene expression. Then, Z-scores of *hnRNPL*, *PTBP1*, and *KHDRBS1* gene expression were calculated in each sample and one-way-ANOVA, with Dunn’s multiple comparisons test correction, was performed to evaluate significant differences between the groups (13). Comparison of enriched motif within NEK2-regulated cassette exons with the compendium of RNA-binding motif from (37) were performed using Tomtom Motif comparison tools from the MEME Suite Collection (RRID:SCR_001783) (38, 39). Gene ontology (GO) analysis was performed as previously described, using topGO (RRID:SCR_014798) Bioconductor package, ranking and analyzing ontologies using the elim method (40).

### Extraction of RNA, RT-PCR, and Real-Time PCR Analysis

RNA extraction, RT-PCR, and real-time PCR analysis were performed as previously described (25). All primers used are listed in Additional File 1: **Supplementary Table 2**.

### Protein Extracts, Co-Immunoprecipitation, and Western Blot Analysis

Total cell extracts were obtained by lysis in 50 mM HEPES, 10 mM MgCl_2_,100 mM NaCl, 10 mM β-glycerophosphate, 2 mM EGTA, 10% (v/v) glycerol, and 1% (v/v) Triton X-100 for HEK293T and in RIPA buffer for MDA-MB-231, as described (25, 41). Nuclear extracts from MDA-MB-231 cells for co-immunoprecipitation were obtained as described (42). Briefly, cells were lysed in 10 mM Hepes, pH 7.9, 1.5 mM MgCl_2_, 10 mM KCl, 1 mM DTT, phosphatase, and protease inhibitors and incubated on ice for 15 min. Next, following addition of NP-40 to a final concentration of 0.6%, cells were vortexed for 10 s and centrifuged 5 min at 16,000 *g* at 4°C. Supernatant was collected as cytoplasmic extract, and the nuclear pellet was lysed in 20 mM HEPES, pH 7.9, 15 mM MgCl_2_, 0.42 M KCl, 0.2 mM EDTA, and 25% (v/v) glycerol and agitated for 30 min at 4°C. After centrifugation for 10 min at 16,000 *g* at 4°C, supernatant was collected as nuclear fraction. Lysates were diluted with 20 mM HEPES, pH 7.9, 15 mM MgCl_2_, 0.2 mM EDTA, and 25% (v/v) glycerol and incubated overnight at 4°C with NEK2 antibody (Santa Cruz, RRID:AB_1126558) or control mouse IgG. Protein-G magnetic beads (Dynabeads, Invitrogen) were added and the sample was incubated at 4°C for 3 h. Beads were washed five times with 20 mM HEPES, pH 7.9, 15 mM MgCl_2_, 120 mM KCl, 0.2 mM EDTA, and 25% (v/v) glycerol, denatured with SDS-sample buffer, and analyzed by SDS-PAGE. Western blot was performed as previously described (18, 25, 35). Nuclear extracts and immunoprecipitation from HEK293T cells were obtained as previously described (43), using either anti-FLAG antibody (Sigma Aldrich, RRID:AB_259529) or control mouse IgG. Western blot analysis was carried out using the following primary antibodies: anti-NEK2 (Santa Cruz, RRID:AB_1126558), anti-ACTIN (Santa Cruz, RRID:AB_2714189), anti-MYC epitope (Santa Cruz, RRID:AB_2857941), anti-MYC (Cell Signaling, RRID:AB_2151827), anti-GAPDH (1:1000 RRID:AB_627679), anti-HSP90 (Santa Cruz, RRID:AB_675659), anti-hnRNPL (Sigma Aldrich, RRID: AB_261966), anti-FLAG (Sigma Aldrich, RRID:AB_259529), rabbit anti-SAM68 (Santa Cruz, RRID:AB_631869), anti-GFP (Santa Cruz, RRID:AB_627695), anti-ERK2 (Santa Cruz, RRID:AB_2141292), and goat anti-PTBP1 (Santa Cruz, RRID:AB_2253470).

### Immunokinase Assays

Anti-MYC antibody (1 µg) (Santa Cruz, RRID:AB_2857941) was incubated for 1 h, with a mixture of protein A/G-Sepharose beads (Sigma-Aldrich) in PBS/0.05% BSA, under constant shaking at 4°C. At the end of the incubation, the beads were washed twice with PBS/0.05% BSA, twice with lysis buffer, and then incubated for 90 min at 4°C with the HEK293T cell extracts (0.5 mg of protein) under constant shaking. Sepharose bead-bound immunocomplexes were rinsed three times with lysis buffer and washed twice with NEK2-kinase buffer (50 mM HEPES, pH 7.5, 5 mM glycerophosphate, 5 mM MnCl_2_, 5 mM NaF, 0.1 mM sodium orthovanadate, 1 mM DTT, and protease inhibitors). Kinase reactions were carried out in 50 µl for 20 min at 30°C in kinase buffer supplemented with 10 µM [^32^P]-ATP (0.2 µCi/µl), 4 µM ATP, 1 µg of cAMP-dependent protein kinase inhibitor, and the appropriate substrate (GST-Sam68 N-term or C-term). Reactions were stopped by adding SDS-sample buffer and analyzed by SDS-PAGE and autoradiography.

### Wound-Healing and Cell-Invasion Assays

Control or SAM68 silenced MDA-MB-231 were seeded at 100% of confluence into ibidi Culture inserts to create a cell-free gap on the dish. Following two washes with PBS and addition of 1% FBS supplemented medium, inserts were removed and the plate was photographed immediately and every hour for 12 h. Area quantification of the gap was performed with ImageJ software using the MRI Wound Healing tool. For cell invasion assay, MDA-MB-231 cells were seeded into the IncuCyte Clearview 96-well inserts (Sartorius; 1,000 cells/well). Insert membrane had been pre-coated on both sides with 50 μg/ml Matrigel (Corning), diluted in RPMI 1640. Lower chambers were filled with 200 μl of either chemotaxis assay medium (RPMI 1640, supplemented with 10% FBS) or negative control medium (RPMI 1640, without FBS). Images were acquired with IncuCyte SX5 Live-content imaging system every hour for 24 h at 10× magnification. Migrated cells were quantified using the IncuCyte Chemiotaxis migration software (phase-contrast; top/bottom), starting 2 h after initial seeding to allow settlement of cells. In both assays, NEK2-chemical inhibition was achieved by treatment with 3 µM JH295 (44).

### Quantification and Statistical Analysis

Statistical analyses for qPCR, densitometric analysis of PCR, and migration and invasion assays were performed in GraphPad Prism (RRID:SCR_002798) according to the statistical tests described in the figure legends. Number of replicates independently analyzed is indicated by the “*n*” in each figure legend. Results were considered significant if *p*-value ≤ 0.05.

## Results

### NEK2 Interacts With Splicing Factors in TNBC Cells

**Figure 1:**
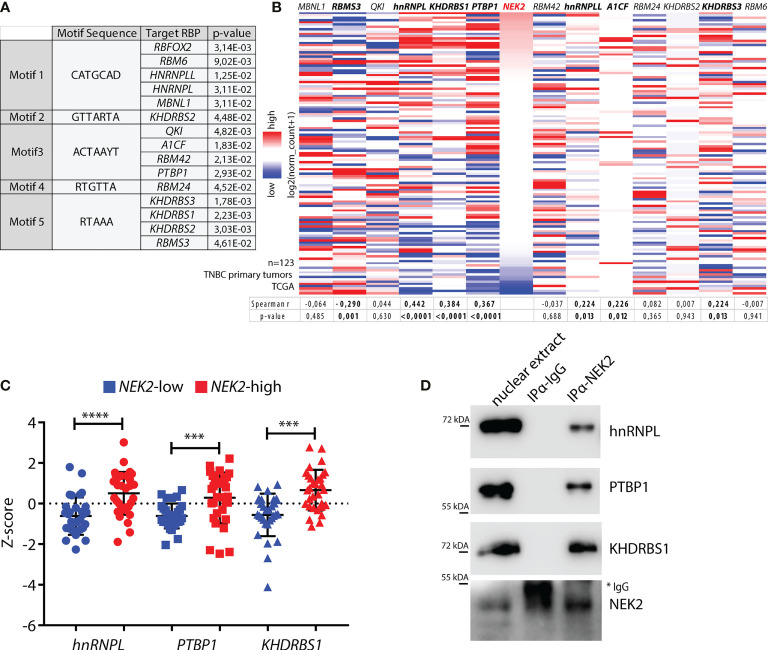
NEK2 is co-expressed and interacts with select splicing factors in TNBC. **(A)** Table showing motifs enriched in sequences of NEK2-regulated cassette exons in MDA-MB-231 cells (GSE140803) and their putative cognate RNA-binding proteins (RBP), identified by the Tomtom motif comparison tool. Only significant results retrieved by the tool are shown (*p*-value ≤ 0.05). **(B)** Heatmap showing expression levels of NEK2 and indicated RBP in primary triple-negative breast cancer (TNBC), according to analysis of transcriptomic data of TCGA project using the UCSC Xena platform. Spearman correlation factors and *p*-value between the expression levels of *NEK2* and every RBP are indicated in the table below the heatmap. **(C)** Expression profile for indicated RBP according to TCGA transcriptomic data of TNBC patients, classified according to Z-score normalization in *NEK2*-low (blue points) and *NEK2*-high (red points) expressing groups. Mean and ± SD are shown in the dot plot. Statistical significance was calculated by one-way ANOVA, with Dunn’s multiple comparisons test, ***p < 0.001, ****p < 0.0001. **(D)** Western blot analysis for indicated RBPs for co-immunoprecipitation assay of NEK2 and control IgG in nuclear extracts from MDA-MB-231 cells. * marks IgG used for immunoprecipitation.

We recently found that NEK2 exerts widespread modulation of the alternative splicing program of MDA-MB-231 (GSE140803), a cell line representative of the TNBC subtype (25). A substantial fraction of the NEK2-regulated splicing events were dependent on the ability of the kinase to promote the expression of RBFOX2 (25), a splicing factor involved in the regulation of the epithelial-to-mesenchymal transition (EMT) process (25). However, other exons regulated by NEK2 lacked binding sites for RBFOX2 and were likely regulated by other splicing factors in TNBC cells. Moreover, NEK2 shows an increased localization in the nucleoplasm and chromatin-bound fraction of TNBC cells, suggesting that it might also physically interact with splicing factors and regulate their activity. To test this hypothesis, we searched for splicing factors that can bind the 5 sequence motifs enriched in the NEK2-regulated cassette exons (25). Computational analyses using the Tomtom Motif comparison tool (38, 39) identified 14 splicing factors that might bind to these sequence motifs (**Figure 1A**), including the already characterized RBFOX2 (25). Next, to evaluate which of these factors could functionally interact with NEK2 in TNBC, we assessed whether they are co-expressed with *NEK2* in primary tumors. Query of transcriptomics data from TNBC tumors deposited in The Cancer Genome Atlas (TCGA) database (45) revealed that expression of *hnRNPL*, *PTBP1* (also known as *hnRNP I*), and *KHDRBS1* (also known as *Sam68*) exhibit the highest and most significant positive correlation with *NEK2* expression (**Figure 1B**). Expression of *A1CF*, *KHDRSB3*, and *hnRNPLL* was also positively correlated with that of *NEK2*, albeit to a lesser extent, whereas *RBMS3* expression was negatively correlated (**Figure 1B**). On the other hand, expression of *MBNL1*, *QKI*, *RBM24*, *KHDRSB2*, *RBM42*, and *RBM6* was not correlated with that of *NEK2* (**Figure 1B**). Furthermore, Z-score classification of patients for low and high expression of *NEK2* confirmed that *hnRNPL*, *PTBP1*, and *KHDRBS1* levels are significantly higher in the *NEK2*-high group compared to the *NEK2*-low group (**Figure 1C**). Importantly, co-immunoprecipitation experiments using nuclear extracts from MDA-MB-231 revealed that NEK2 physically interacts with hnRNPL, PTBP1, and KHDRBS1 proteins (**Figure 1D**), whereas no interaction was detected for an uncorrelated factor like QKI (**Supplementary Figure 1A**). These findings indicate that the interaction of NEK2 with specific splicing factors could be functionally relevant to modulate the splicing signature of TNBC cells.

### Oncogenic Transcription Factor MYC Drives NEK2 Expression in TNBC

**Figure 2:**
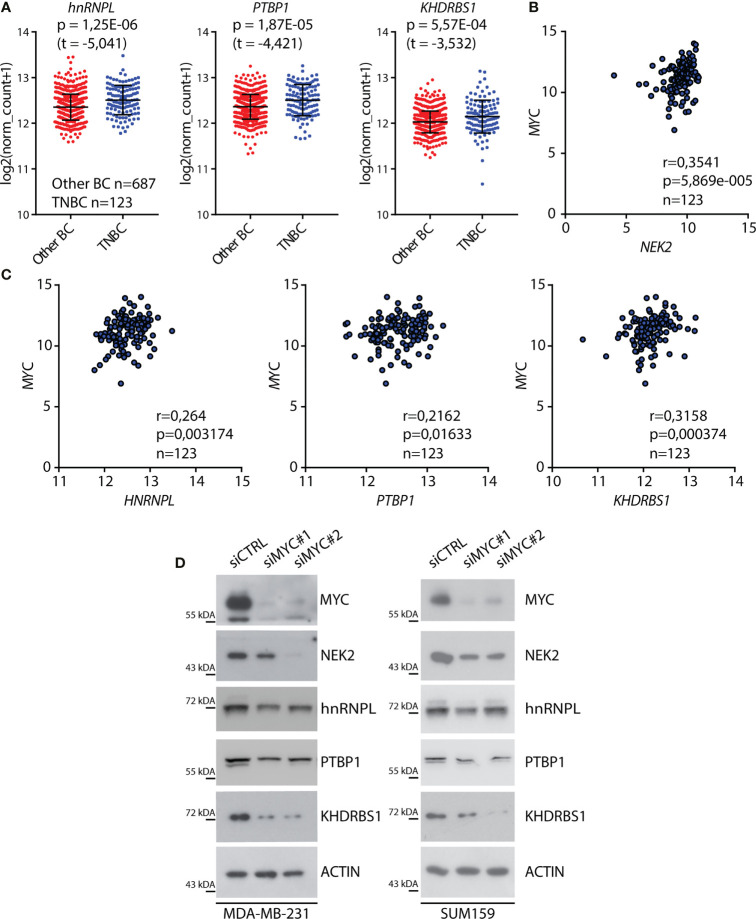
C-MYC regulates the expression of NEK2 in TNBC cells. **(A)** Dot-blot showing expression levels of indicated RNA-binding proteins (RBP) in primary triple-negative breast cancer (TNBC) and other breast cancer (Other BC) subtypes, according to analysis of transcriptomic data of TCGA project using the UCSC Xena platform. Mean and ± SD are shown in the dot plot. Statistical significance was calculated by Welch’s *t*-test. **(B, C)** Scatter plots of RNA expression levels of *MYC* and *NEK2* **(B)** or *MYC* and *hnRNPL, PTBP1*, and *KHDRBS1* **(C)** in primary TNBC according to TCGA data. Spearman’s correlation coefficient (*r*) and associated *p*-value are shown. **(D)** Representative Western blot analysis for MYC, NEK2, hnRNPL, PTBP1, and KHDRBS1 expression levels in MDA-MB-231 (left panel) and SUM159 (right panel) cells, transiently transfected with indicated siRNAs. ACTIN was evaluated as loading control.

Increased nuclear localization of NEK2 in TNBC is driven by its higher expression levels compared to other BC subtypes (25). Since we identified splicing factors that are co-expressed and interact with NEK2 in this tumor subtype, we asked if a common transcription factor could promote their expression. In particular, we focused our attention on the proto-oncogenic transcription factor c-MYC, which is overexpressed in TNBC compared to other BC subtypes (**Supplementary Figure 1B**) (46) and was shown to drive transcription of both *NEK2* (32) and its putative cofactors *PTBP1* (12) and *KHDRBS1* (13) in other tumoral context. Analysis of expression data in the TCGA database revealed a significant upregulation of *hnRNPL*, *PTBP1*, and *KHDRBS1* expression in TNBC compared to other BC subtypes (**Figure 2A**) as previously reported for *NEK2* (25). In addition, we observed that *MYC* expression was positively correlated with the expression of *NEK2* (**Figure 2B**) and of its putative co-factors *hnRNPL*, *PTBP1*, and *KHDRBS1* (**Figure 2C**). By contrast, no significant correlation was observed between *MYC* and *QKI* expression (**Supplementary Figure 1C**), whose expression is not correlated with *NEK2* (**Figure 1B**). These observations suggest that c-MYC could coordinate the expression of NEK2 and its interacting splicing factors in TNBC. To test this hypothesis, we asked whether c-MYC silencing affects the expression of these proteins in TNBC cells. Western blot analyses of extracts from MDA-MB-231 and SUM159 transiently transfected with two different c-MYC siRNAs revealed that c-MYC depletion reduced the expression of NEK2, hnRNPL, PTBP1, and KHDRBS1 in both TNBC cell lines (**Figure 2D**). Collectively, these observations suggest that c-MYC overexpression sustains the concomitant expression of NEK2 and select splicing factors in TNBC cells, thereby favoring their interaction.

### Sam68 Is a Direct Substrate of NEK2 Kinase

**Figure 3:**
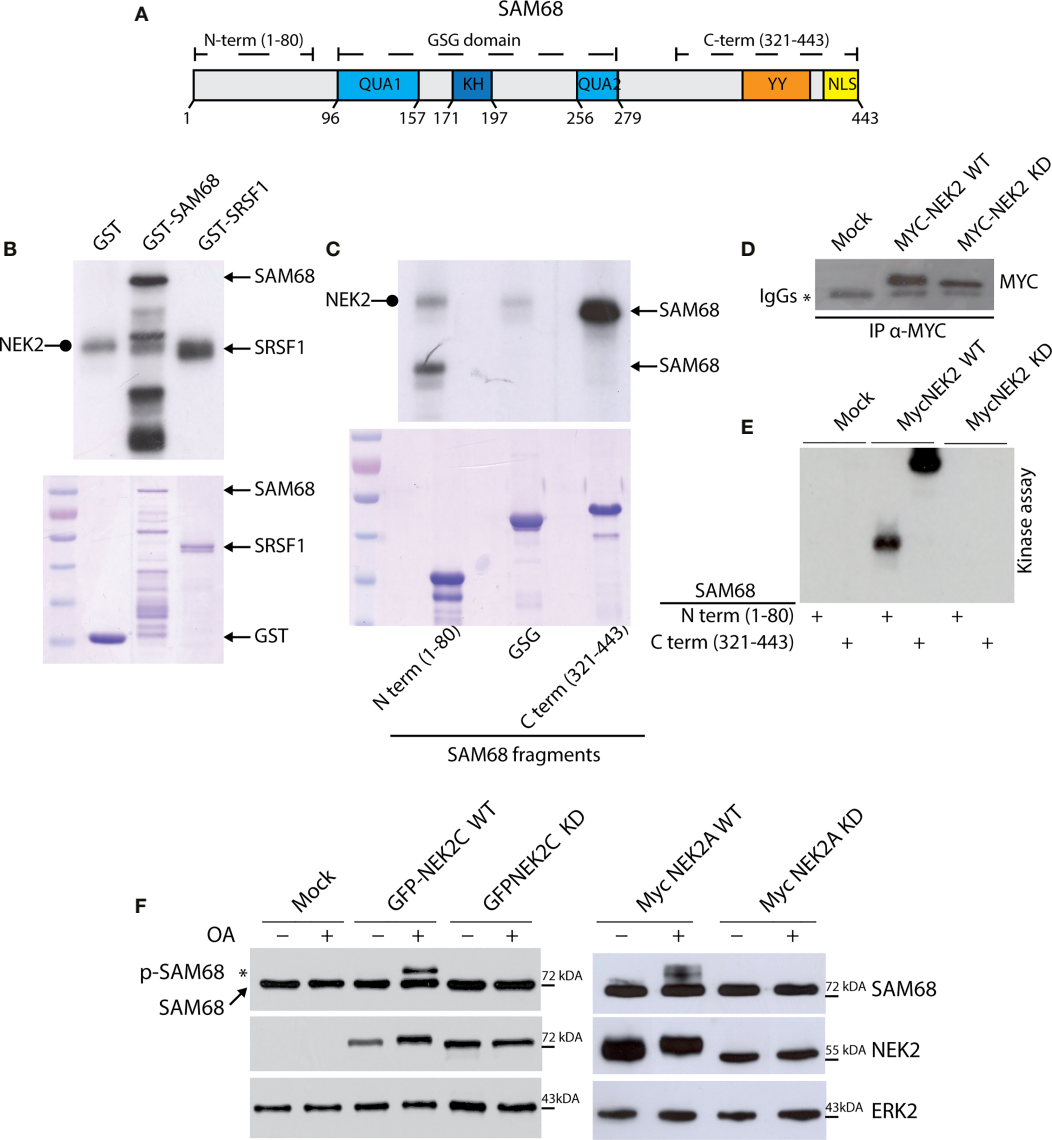
NEK2 phosphorylates SAM68 *in vitro*. **(A)** Schematic representation of protein domain in human full-length SAM68 protein. **(B)** Representative autoradiography for NEK2 kinase assay, performed by incubating an active purified-NEK2 protein with recombinant GST and a full-length SAM68 and SRSF1 as substrates. Coomassie staining was performed as loading control. Rounded tip arrows indicate auto-phosphorylated NEK2. **(C)** Representative autoradiography and Coomassie staining for a kinase assay performed by incubating an active purified-NEK2 recombinant GST N-terminal, GSG domain or C-terminal of SAM68 as substrates. **(D, E)** Representative Western blot analysis **(D)** and autoradiography **(E)** for immunokinase assay, performed by incubating immunoprecipitated wild-type NEK2 (WT) or kinase-dead NEK2 (KD) with recombinant N-terminal or C-terminal GST-Sam68 as substrate. **(F)** Western blot analysis for MYC-Sam68 protein in total extracts from HEK293T cells transfected with expression vectors for GFP-tagged NEK2C wild-type (WT) or kinase-dead (KD), or with MYC-tagged NEK2A variant WT or KD. Tag-specific antibodies were used for recombinant NEK2 detection. ERK2 was evaluated as loading control. Activation of NEK2 was obtained by treating cells with 0.1 μM OA for the last 3 h before collection. * marks the molecular weight shift in SAM68 protein elicited by OA-mediated activation of WT NEK2C (left panel) and WT NEK2A (right panel).

To functionally test the interaction between NEK2 and splicing factors in TNBC, we focused on KHDRBS1, hereafter named Sam68 (**Figure 3A**), because its activity is extensively modulated by phosphorylation (43, 47–50). Moreover, Sam68 was more dependent on MYC expression than hnRNPL and PTBP1 in both TNBC cell lines tested (**Figure 2D**). First, we confirmed the physical interaction between the proteins by co-immunoprecipitation of transiently transfected FLAG-Sam68 and GFP-NEK2 in HEK293T cells (**Supplementary Figure 2**). Next, we performed *in vitro* kinase assays in the presence of labeled ATP ([γ-32P]ATP) using purified recombinant NEK2 and GST-Sam68. NEK2 readily phosphorylated GST-Sam68, to a similar extent of its known substrate GST-SRSF1 (18), whereas GST alone was not phosphorylated under the assay conditions (**Figure 3B**). Sam68 comprises an hnRNP K homology (KH) RNA binding motif flanked by the QUA1 and QUA2 motifs, which form the GRP33/Sam68/GLD1 (GSG) domain required for dimerization and high affinity RNA binding, and regulatory regions at the N and C terminus that contain sites for protein–protein interactions and post-translational modifications (**Figure 3A**) (47, 48, 51). NEK2 phosphorylates with high efficiency the N-terminal and C-terminal regulatory regions of Sam68, whereas the GSG domain was barely phosphorylated (**Figure 3C**). Moreover, *in vitro* kinase assays using wild type (WT) or kinase-dead (KD) NEK2 immunoprecipitated from transfected HEK293T cells confirmed that the enzymatic activity of NEK2 was directly responsible for phosphorylation of the regulatory regions of Sam68 (**Figures 3D** **, E**).

Next, we asked if NEK2 phosphorylates Sam68 also in live cells. To this end, HEK293T cells were transfected with plasmids encoding MYC-Sam68 and either WT or KD versions of MYC-tagged NEK2A and GFP-tagged NEK2C, a splice variant of the kinase that is predominantly localized in the nucleus like Sam68 (52). Upon treatment with the protein phosphatase 1 (PP1) and 2A (PP2A) inhibitor Okadaic Acid (OA) to elicit NEK2 activation (33), NEK2 induced a shift in the electrophoretic mobility of Sam68 (**Figure 3F**), which is a hallmark of its phosphorylation in serine/threonine residues (49). Notably, the slower migrating form of Sam68 was observed only in cells co-transfected with WT NEK2, but not with the catalytically inactive KD mutant. Furthermore, NEK2C displayed higher ability to induce Sam68 phosphorylation, further supporting a functional interaction in the nucleus between the proteins. These results identify Sam68 as a novel substrate of NEK2.

### NEK2-Mediated Phosphorylation Modulates Sam68 Splicing Activity

**Figure 4:**
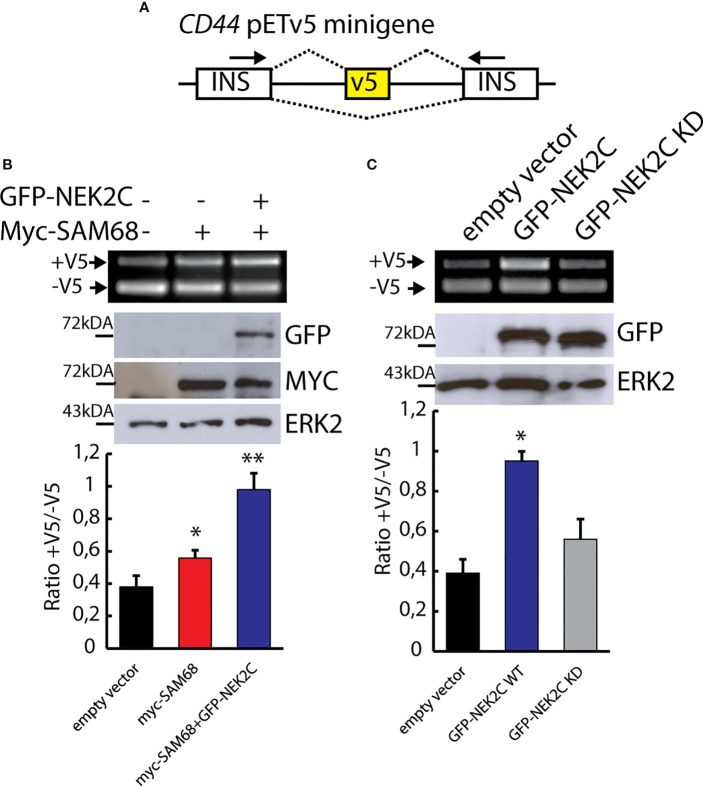
NEK2 phosphorylates Sam68 *in vivo* and modulates its splicing activity. **(A)** Schematic representation of the *CD44* pETV5 minigene. Alternative exon v5 of the *CD44* gene was cloned between two constitutive cassette exons insulin exons 2 and 3. **(B, C)** Representative PCR and Western blot analysis for HEK293T cells transfected with the *CD44* pETV5 minigene and expression vectors for MYC-tagged SAM68 and GFP-tagged NEK2C wild-type (WT) or kinase-dead (KD). Western blot analysis for ERK2 was used as loading control. Densitometric analyses for all experiment were performed and ratio between CD44 (+V5) and CD44 (-V5) is represented by histogram bars (mean ± SD, *n* = 3; *t*-test *p < 0.05, **p < 0.01).

Serine/threonine phosphorylation by the mitogen activated protein kinases (MAPKs) was shown to regulate the splicing activity of Sam68 (16, 49, 50, 53). To investigate whether NEK2-dependent phosphorylation also affects Sam68 activity, we employed a reporter minigene that recapitulates the splicing regulation of the *CD44* v5 exon (pET-V5 minigene) (**Figure 4A**), which is a target of Sam68 and is sensitive to its activation by serine/threonine phosphorylation (49, 54). HEK293T cells were co-transfected with plasmids encoding the pET-V5 minigene, MYC-Sam68, and GFP-NEK2C. As expected, sub-optimal amounts of MYC-Sam68 promoted the inclusion of CD44 variable exon v5 (**Figure 4B**; lane 2). Co-expression of NEK2C significantly enhanced this effect, leading to an almost doubled inclusion of the v5 exon with respect to cells transfected with Sam68 alone (**Figure 4B**; lane 3). Furthermore, u**preg**ulation of NEK2C alone, but not of its cognate KD mutant, was sufficient to promote exon v5 inclusion (**Figure 4C**), suggesting that it might affect the splicing activity of the endogenous Sam68 protein. These results indicate that NEK2-dependent phosphorylation of Sam68 modulates its splicing activity and that their physical interaction might be functionally relevant in TNBC cells.

### Sam68 Modulates TNBC Cell Transcriptome

**Figure 5:**
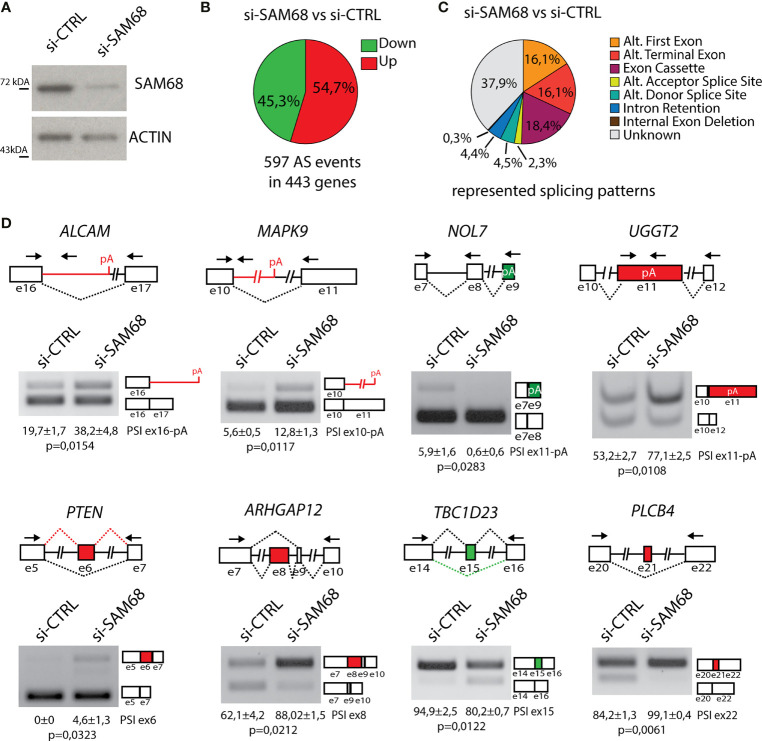
Sam68 regulates alternative splicing in TNBC cells. **(A)** Representative Western blot analysis assessing SAM68 silencing efficiency expression in MDA-MB-231 cells transiently transfected with indicated pool of siRNAs. ACTIN was evaluated as loading control. **(B)** Pie chart showing percentage of upregulated (red) and downregulated (green) exons in the si-SAM68 vs. si-CTRL comparison. **(C)** Pie chart showing percentages of indicated different splicing pattern among regulated splicing events in the si-SAM68 vs. si-CTRL comparison. **(D)** Representative PCR analysis for indicated alternative splicing events in si-SAM68 vs. si-CTRL MDA-MB-231 cells. Schematic representation for each event analyzed is depicted below relative agarose gels. Green and red boxes indicate down- and upregulated exons in si-SAM68 vs. si-CTRL cells. Percentage of splicing inclusion (PSI) of indicated exons was evaluated by densitometric analysis, and results are shown below agarose gels (mean ± SD, *n* = 3, *t*-test).

Sam68 is upregulated in breast tumors compared to normal tissue and promotes BC cell proliferation (55). However, although the oncogenic function of Sam68 has been often related to its splicing activity (47, 50, 53), genome-wide characterization of its splicing targets in BCs or other cancer types is still lacking. To elucidate the splicing signature regulated by Sam68 in TNBC cells, we carried out RNA-sequencing (RNA-seq) analyses of MDA-MB-231 cells that were transiently depleted of Sam68 (**Figure 5A**). Bioinformatics analyses using the reference FAST-DB database (25, 35, 41), revealed a large modulation of the TNBC cell transcriptome by Sam68, with 443 genes regulated at splicing level and 474 at gene expression level upon its depletion (**Figure 5B**; **Supplementary Figure 3A**; Additional File 2: **Supplementary Tables 1, 2**). More than half of the 597 regulated exons (54,7%) are u**preg**ulated in Sam68-depleted cells, suggesting that Sam68 preferentially functions as a splicing repressor in TNBC cells. Classification of the regulated splicing events revealed that exon cassettes (18.4%) and alternative terminal exons (16.1%) are the most regulated patterns (**Figure 5C**). Importantly, RT-PCR analysis of 16 of these splicing events using an independent set of control and Sam68-depleted MDA-MB-231 cells confirmed the RNA-seq results (**Figure 5D**; **Supplementary Figure 3B**), thus validating the reliability of the bioinformatics analyses. These results show that Sam68 significantly contributes to the splicing signature of TNBC cells.

### Sam68 and NEK2 Co-Regulate Alternative Splicing Events in TNBC Cells

**Figure 6:**
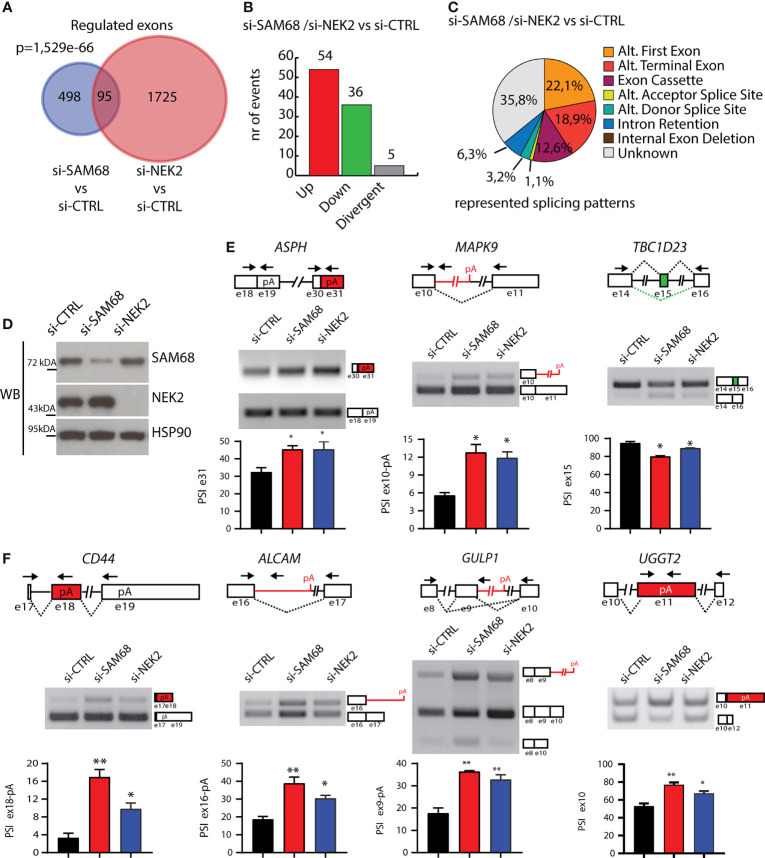
Sam68 and NEK2 co-regulates AS in TNBC cells. **(A)** Venn diagram showing the overlap between regulated alternative exons in MDA-MB-231 cell silenced for either SAM68 (this study) or NEK2 (GSE140803). **(B)** Bar graph showing the number of splicing events either divergently (gray bar) or commonly upregulated (red bar) or downregulated (green bar) in si-SAM68 and si-NEK2 MDA-MB-231 cells compared to control. **(C)** Pie chart showing percentages of indicated splicing patterns among the common splicing events regulated in the si-SAM68/si-NEK2 vs. si-CTRL comparison. **(D)** Representative Western blot analysis assessing SAM68 and NEK2 silencing efficiency in MDA-MB-231 cells transiently transfected with indicated pool of siRNAs. HSP90 was evaluated as loading control. **(E, F)** Representative PCR analysis for indicated alternative splicing events in si-CTRL, si-SAM68, and si-NEK2 MDA-MB-231 cells. Schematic representation for each event analyzed is depicted besides relative agarose gels. Green and red boxes indicate commonly down- and upregulated exons in si-SAM68/si-NEK2 vs. si-CTRL cells. Bar graphs below each agarose gel represent percentage of splicing inclusion (PSI), evaluated by densitometric analysis (mean ± SD, *n* = 3, one-way ANOVA, *p < 0.05, **p < 0.01).

Next, we asked whether NEK2 modulates the splicing activity of the endogenous Sam68 in TNBC cells. To this end, we compared the splicing signatures of Sam68-silenced (**Figures 5B, C**) and NEK2-silenced MDA-MB-231 cells (25). We found a significant overlap between the two datasets, with 95 alternative splicing events that are commonly regulated by Sam68 and NEK2 depletion (**Figure 6A**). Annotation of these events revealed that most of them were modulated in the same direction by silencing of either Sam68 or NEK2 (**Figure 6B**). Nearly half of the splice variants commonly regulated by Sam68 and NEK2 were novel transcripts originating from either unannotated splicing events or selection of an alternative transcription start site, while alternative last exon and exon cassette were the predominant splicing patterns among the remaining events (**Figure 6C**). RT-PCR analysis using RNA from an independent set of samples confirmed that depletion of Sam68 or NEK2 regulated a common pattern of splicing in three of these genes (*ASPH*, *MAPK9*, and *TBC1D23*) in MDA-MB-231 cells (**Figures 6D, E**). Furthermore, RT-PCR analyses revealed that additional Sam68 target exons, like those in *ALCAM*, *CD44*, *GULP1*, and *UGGT2* genes, were also sensitive to NEK2 depletion in MDA-MB-231 (**Figure 6F**), even though they were not highlighted by the bioinformatics analysis (25). These results indicate that Sam68 and NEK2 share common splicing targets in TNBC cells and further suggest their functional interaction in splicing regulation in this tumor type.

### Sam68 and NEK2 Cooperate in the Regulation of TNBC Cell Migration and Matrix Invasion

**Figure 7:**
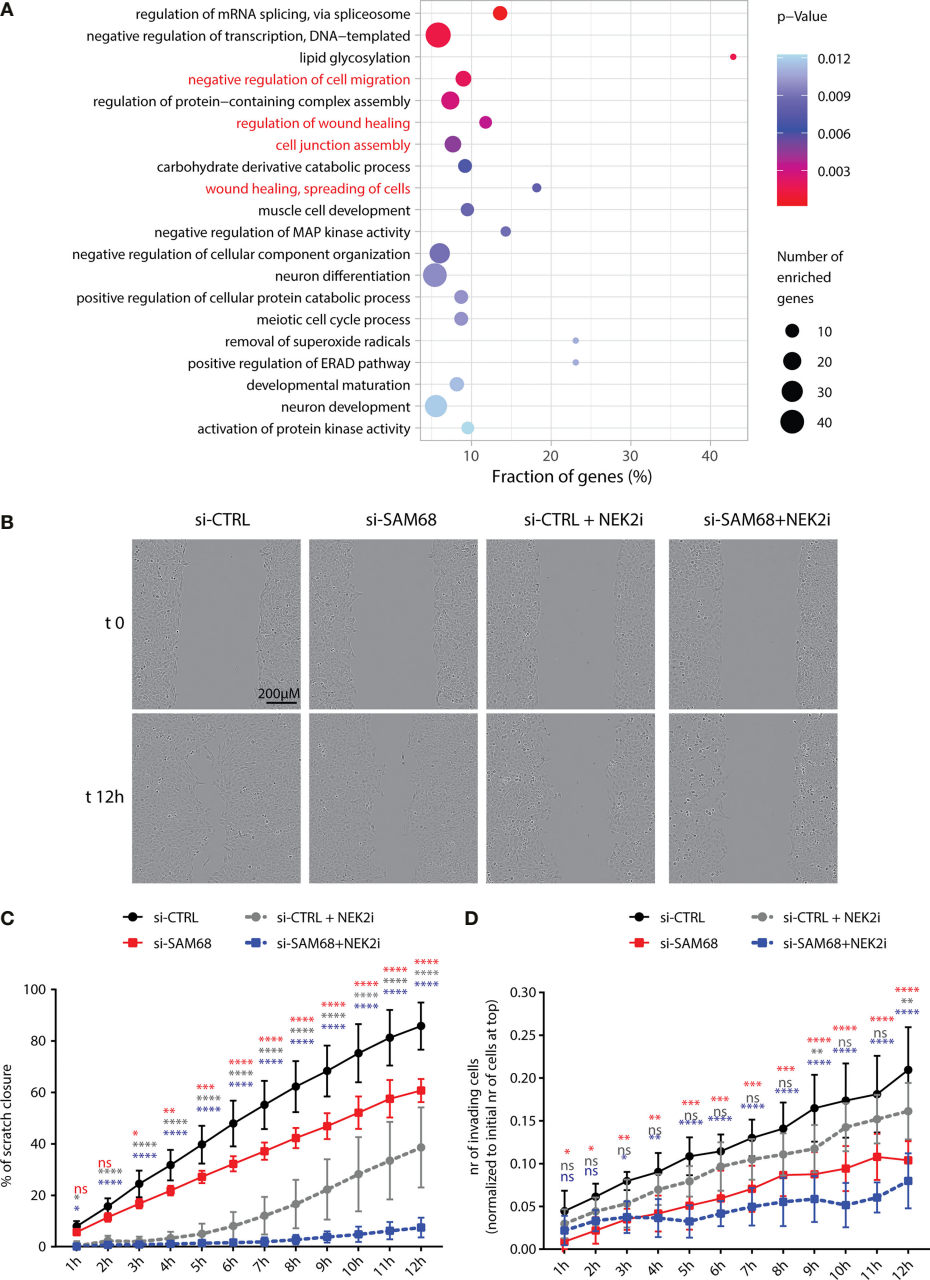
Sam68 and NEK2 co-regulate cell migration in TNBC cells. **(A)** Gene ontology analysis of biological process of AS regulated genes in the comparison between control and Sam68 silenced MDA-MB-231 cells (*p*-value ≤ 0.05). **(B)** Representative micrograph images, at the initial time point (t0) and 12 h later (t12) of the wound-healing assay performed on control (si-CTRL) or SAM68 silenced (si-SAM68) MDA-MB-231 cells, treated or not with the NEK2 inhibitor (NEK2i) JH295 [3 µM]. **(C)** Line graph showing the percentage of wound closure of si-CTRL and si-SAM68 MDA-MB-231 cells, treated or not with NEK2i (mean ± SD, *n* = 6, *p < 0.05, **p < 0.01, ***p < 0.001, ****p < 0.0001, ns = not significant, two-way ANOVA, colors indicate the growth condition to whom si-CTRL cells were compared in the statistical analysis). **(D)** Line graph showing the number of si-CTRL and si-SAM68 MDA-MB-231 cells, treated or not with NEK2i, invading Matrigel-coated transwell of the IncuCyte Clearview 96-well insert system. Number of invading cells on the bottom side of the insert at every hour was normalized to the initial number of cells on the top of the insert at initial seeding (mean ± SD, *n* = 6, *p < 0.05, **p < 0.01, ***p < 0.001, ****p < 0.0001, ns= not significant, two-way ANOVA, colors indicate the growth condition to whom si-CTRL cells were compared in the statistical analysis).

GO analysis of the Sam68 splicing-regulated genes highlighted a significant enrichment for terms related to biological processes involved in cell adhesion and migration (**Figure 7A**). Moreover, genes related to the wound-healing process were enriched among the common targets of Sam68 and NEK2 (Supplementary **Figure 4**). These process are frequently deregulated in TNBC and contribute to their aggressive and metastatic phenotype (27, 56). Thus, we asked if Sam68 ablation could affect these pro-metastatic functions. Wound-healing and matrigel-invasion assays revealed that Sam68 depletion caused a significant impairment of the migratory and invasive properties of MDA-MB-231 cells (**Figures 7B** **–D**). Notably, we also found that the effects elicited by Sam68 knockdown were worsened by concomitant treatment of MDA-MB-231 cells with JH295, an irreversible inhibitor of NEK2-kinase activity (**Figures 7B** **–D**) (44). Collectively, these observations indicate that the functional interaction with NEK2 potentiates the splicing activity of Sam68 and enhances the motility and invasive properties of TNBC cells.

## Discussion

Alternative splicing dysregulation is a common trait of human cancers, which affects multiple cellular processes in the course of tumorigenesis (2, 3, 17). Thus, characterization of the molecular mechanisms underlying aberrant splicing offers the opportunity to identify new targets for cancer therapy. This issue is particularly interesting for TNBC, as these tumors currently lack targeted and efficacious therapies, but features a specific splicing signature (8, 10, 25). In this regard, targeting either the expression of specific splicing factors or inhibiting the spliceosome activity was shown to selectively halt TNBC cell proliferation (57). We have previously reported that the mitotic kinase NEK2 is upregulated in TNBC with respect to other BC types and promotes a specific pro-mesenchymal splicing program that confers metastatic features to TNBC (25). Herein, we found that NEK2 interacts with select splicing factors in TNBC cells and, as indicated by its functional interaction with Sam68, could enhance their splicing activity and oncogenic functions.

NEK2 is highly expressed in primary TNBC along with Sam68, hnRNPL, and PTPBP1, whose cognate binding motifs are enriched in NEK2-sensitive cassette exons and physically interact with this kinase. Moreover, knockdown of Sam68 (this study) and hnRNPL (25) partially recapitulated the splicing changes observed in TNBC cells depleted of NEK2, suggesting that this kinase modulates splicing through functional interaction with splicing factors in TNBC cells. We also found that expression of NEK2 and its interacting splicing factors in primary TNBC correlates with that of MYC, suggesting that this transcription factor coordinates a splicing network that contributes to the TNBC-specific splicing signature. In support of this hypothesis, MYC depletion in representative TNBC cell lines caused reduced expression of NEK2, Sam68, hnRNPL, and PTBP1 proteins. MYC is a powerful oncogene and is highly expressed in TNBC compared to other BCs (46). Moreover, MYC upregulation was shown to impose a transcriptional stress to cancer cells that increases their dependency on the proper functionality of the splicing machinery (58). Thus, it is tempting to speculate that coordination of the expression of NEK2 and its interacting splicing factors represents a pro-survival mechanism that is selected in MYC-driven TNBC to cope with such transcriptional/splicing stress. In this view, targeting NEK2 expression and/or activity could represent a therapeutic vulnerability for MYC-driven TNBC, as previously shown for inhibition of the spliceosome (58). Remarkably, MYC regulates transcription of other splicing factors (SRSF1 and hnRNPA1) that interact with and are regulated by NEK2 in other tumoral contexts (12, 14, 18, 32). Thus, NEK2 inhibition could represent an exploitable vulnerability also for other types of MYC-driven tumors.

Sam68 is a multifunctional RBP, whose splicing activity exerts a pivotal role for the proper differentiation of neuronal and germ cells (35, 59, 60). Notably, although several studies have shown the oncogenic activity of Sam68 in different human cancers (47, 48), a global analysis of the regulation exerted by this protein on the human transcriptome was still lacking. Herein, genome-wide RNA-seq analysis identified hundreds of splicing events modulated by Sam68 depletion in TNBC cells. Similarly to previous observations in *Sam68* knockout mice (35, 59), exon cassettes and alternative terminal exons were the most affected splicing patterns in MDA-MB-231 cells. Of note, functional annotation of the splicing-regulated genes revealed enrichment for terms relative to neuronal and muscular development, as well as to meiosis, all biological processes that are impaired in *Sam68* knockout mice (35, 59–62). These observations are suggestive of an evolutionary conserved splicing program regulated by Sam68, which is hijacked by cancer cells to sustain oncogenic transformation.

Serine/threonine phosphorylation is one of the major post-translational modifications shown to promote the pro-oncogenic splicing activity of Sam68 (49, 50, 53, 63). Most of these studies identified the MAPK/ERK pathway as responsible for Sam68 phosphorylation and activation (49, 50, 53, 63). By identifying Sam68 as a binding partner and direct substrate of NEK2, we provide evidence for an additional cellular pathway modulating Sam68 phosphorylation and oncogenic splicing activity. Interestingly, activation of the MAPK pathway was shown to promote NEK2 activity in male germ cells (34), suggesting the possible synergy between these kinases in the regulation of Sam68 activity. Given the ubiquitous expression of Sam68 and NEK2 and their frequent upregulation in different cancer types (48, 64), their interaction is likely functional also in other tumors. Thus, our study reveals a new regulatory mechanism of Sam68 function, which adds to the various post-translational modifications, such as tyrosine phosphorylation (43) and acetylation (65), and interactions with regulatory partners, such as the transcriptional cofactors SND1 (54) and FBI-1 (66), that modulate its splicing activity in cancer cells, including TNBC cells.

Our studies have also identified a large number of splice variants regulated by Sam68 and NEK2 that are possibly implicated in the regulation of cancer cell motility and invasiveness. Moreover, combined inhibition of Sam68 expression and NEK2 activity cooperated to suppress TNBC cell migration and matrix invasion, suggesting that modulation of the identified splicing program is functionally relevant. Collectively, these results support the key oncogenic role of NEK2 and suggest that NEK2 targeting approaches represent promising therapeutic tools for TNBC treatment, whose efficacy could be amplified by co-targeting the vulnerability induced by splicing dysregulation in cancer cells.

## Data Availability Statement

The datasets presented in this study can be found in the online repository: GEO database. accession number: GSE140754.

## Author Contributions

CN, FB, MP, and CS contributed to conception and design of the study. CN and CS wrote the manuscript. CN, FB, CC, MM, VP, SA, and MP performed the experiments, and analyzed and interpreted data. All authors contributed to manuscript revision, read, and approved the submitted version.

## Funding

VP was supported by a fellowship from the Associazione Italiana Ricerca sul Cancro (23938). This work was supported by grants from the Associazione Italiana Ricerca sul Cancro (IG23416 and MFAG21899) and Breast Cancer Now (Catalyst Grant n. 2018NovPCC1283). Università Cattolica del Sacro Cuore contributed to the funding of this research project and its publication.

## Conflict of Interest

The authors declare that the research was conducted in the absence of any commercial or financial relationships that could be construed as a potential conflict of interest.

## Publisher’s Note

All claims expressed in this article are solely those of the authors and do not necessarily represent those of their affiliated organizations, or those of the publisher, the editors and the reviewers. Any product that may be evaluated in this article, or claim that may be made by its manufacturer, is not guaranteed or endorsed by the publisher.
