# Supplementary material for: Functional Interaction Between the Oncogenic Kinase NEK2 and Sam68 Promotes a Splicing Program Involved in Migration and Invasion in Triple-Negative Breast Cancer
Source: Front Oncol. 2022 Apr 21;12:880654. doi: 10.3389/fonc.2022.880654 (PMC9068942; doi:10.3389/fonc.2022.880654)
Supplement: Supplementary file 1 [file DataSheet_1.docx]

Supplementary Material

# Supplementary Data

## Supplementary Tables

Supplementary Table 1. Sequences of si-RNAs used in this study

Supplementary Table 2. List of sequences of primers used in this study

Supplementary Table 3. List of the 474 Regulated Genes (Fold-change ≥ 1,5; P-Value ≤ 0,05) in the MDA-MB-231 si-SAM68 vs si-CTRL comparison.

Supplementary Table 4. List of the 597 Differentially Regulated Alternative Splicing Events (from 443 Distinct Genes) in the MDA-MB-231 si-SAM68 vs si-CTRL comparison.

## Supplementary Figures


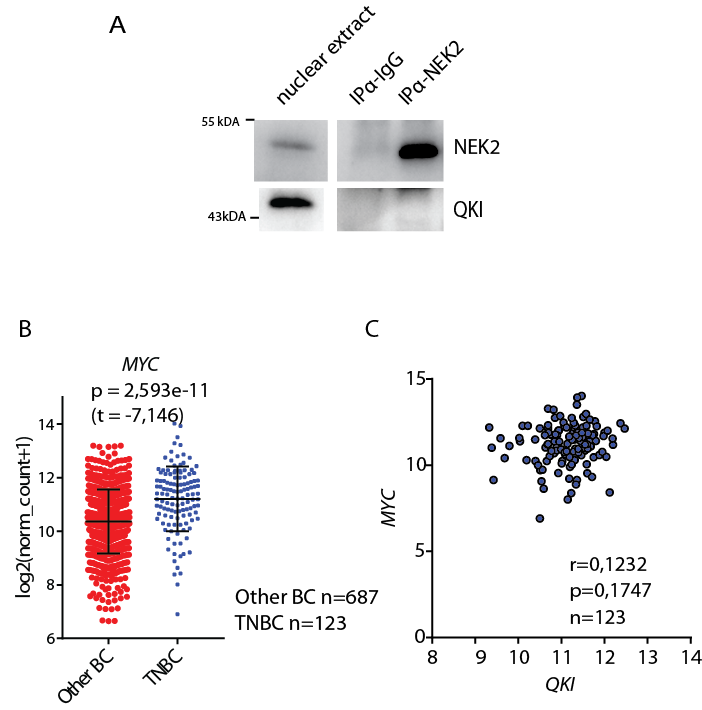


**Supplementary Figure 1. c-MYC is upregulated in TNBC.**

(A) Western-blot analysis of QKI and NEK2 in co-immunoprecipitation assay of NEK2 and control IgG in nuclear extracts from MDA-MB-231 cells. B) Dot-blot showing expression levels of *c-MYC* in primary triple-negative breast cancer (TNBC) and other breast cancer (Other BC) subtypes, according to analysis of transcriptomic data from the TCGA repository. Mean and ± SD are shown in the dot plot. Statistical significance was calculated by Welch’s t-test. (C) Scatter plots of RNA expression levels of *MYC* and *QKI* (C) in primary TNBC according to analysis of transcriptomic data from TCGA. Spearman’s correlation coefficient (r) and associated p-value are shown.

**
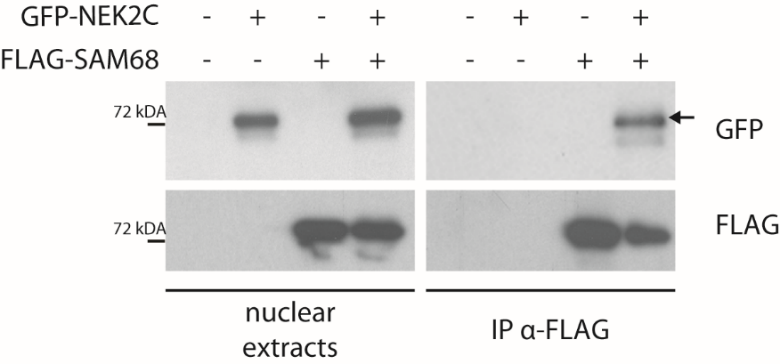
Supplementary Figure 2. NEK2 interacts with SAM68.**

(A) Western blot analysis with GFP- and FLAG- antibodies of co-immunoprecipitation experiments of FLAG-SAM68 in nuclear extracts of HEK293T cells transfected with indicated plasmids. Arrow indicates GFP-NEK2C corresponding band.

**
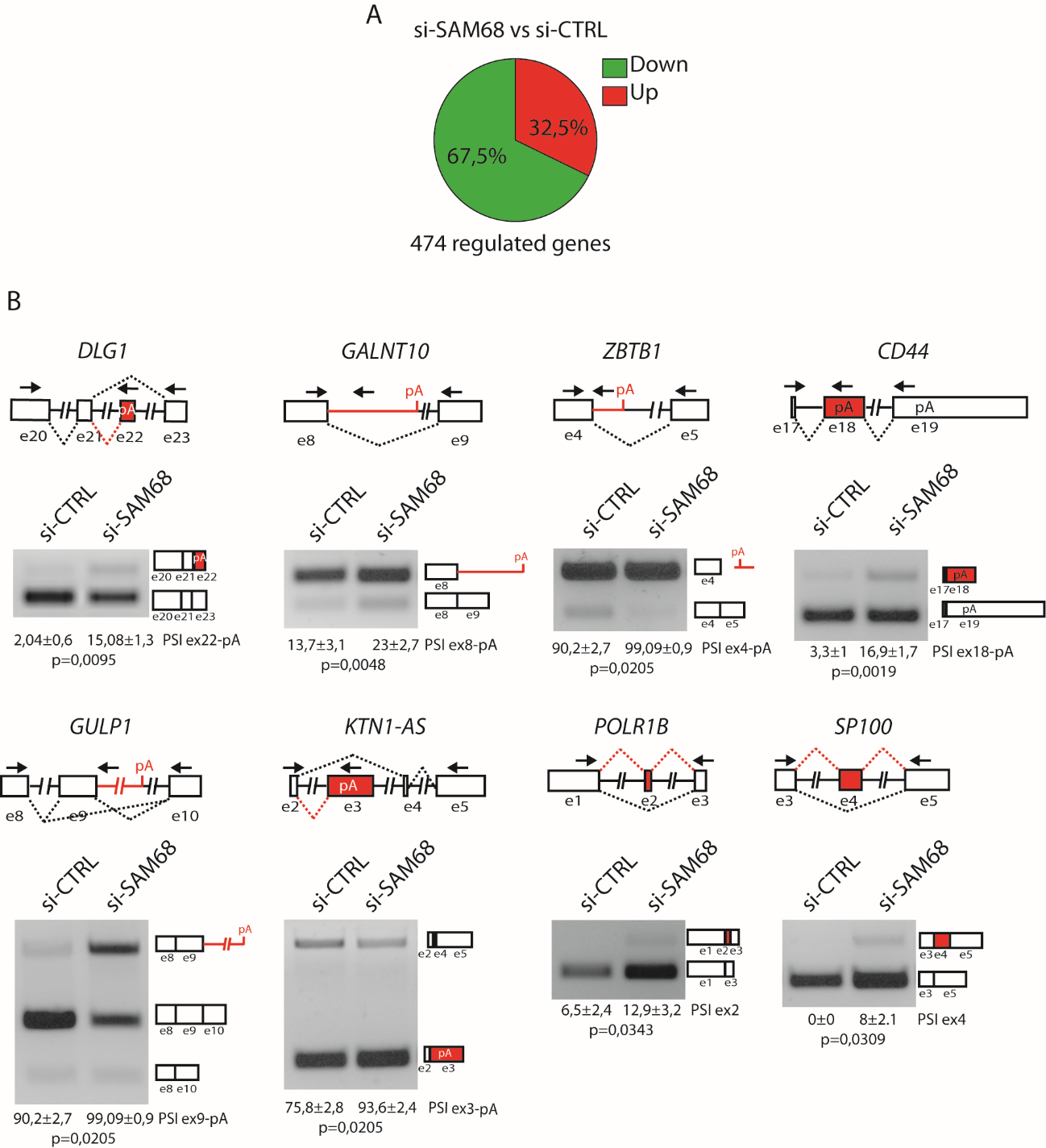
Supplementary Figure 3**. SAM68 depletion modulated TNBC cells transcriptome

(A) Pie chart showing percentages of up- (red) and down- (green) regulated genes at the expression levels, in the SAM68 silenced (si-SAM68) versus control (si-CTRL) MDA-MB-231 cells comparison. (B) Representative PCR analysis for indicated alternative splicing events in si-SAM68 vs si-CTRL MDA-MB-231 cells. Schematic representation for each event analyzed is depicted below relative agarose gels. Green and red boxes indicate down- and up-regulated exons in si-SAM68 vs si-CTRL cells. Percentage of splicing inclusion (PSI) of indicated exons was evaluated by densitometric analysis, and results are shown below agarose gels (mean ± SD, n = 3, t-test, p<0.05 was considered significant).


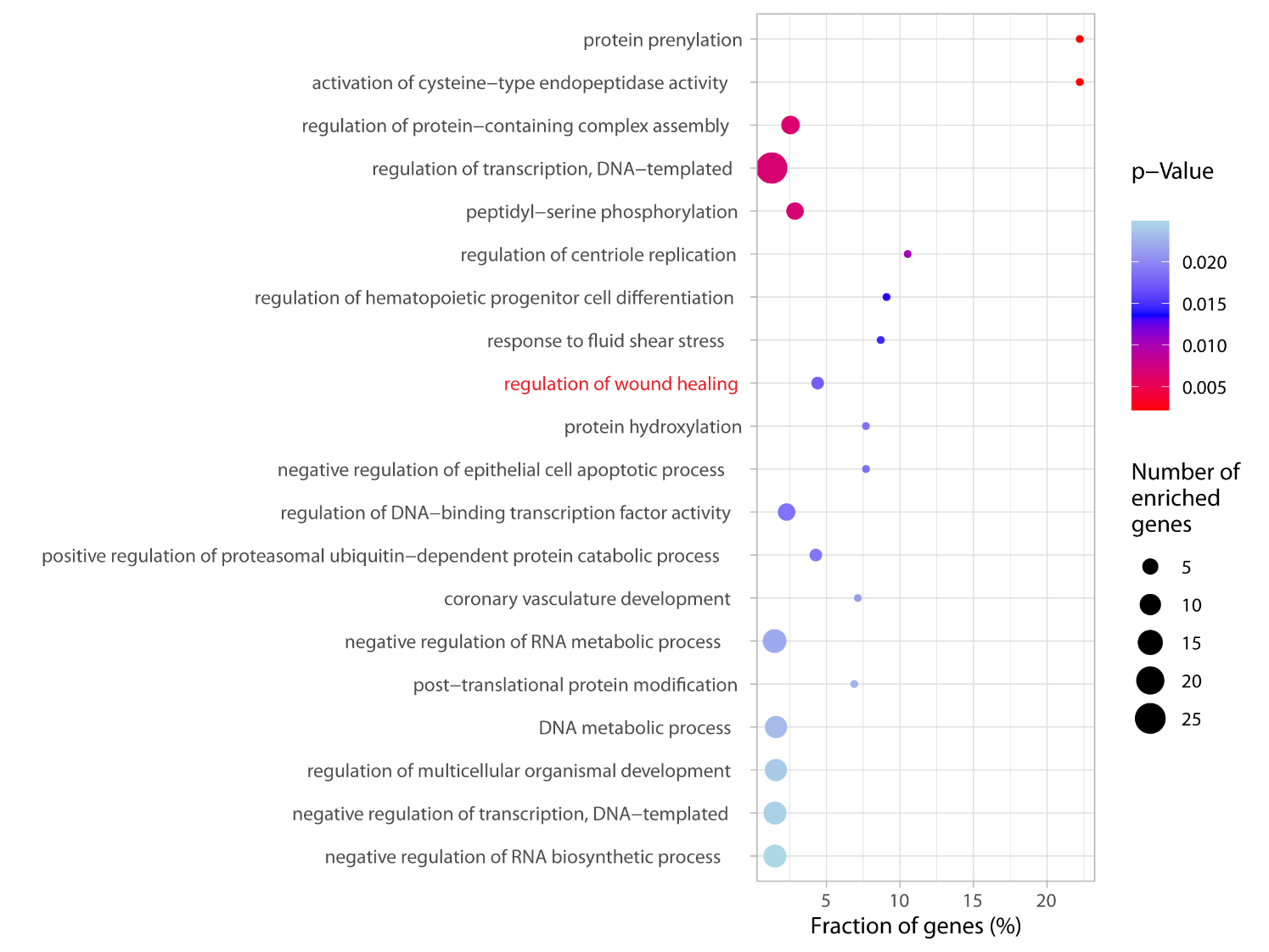


Supplementary Figure 4. Sam68 and NEK2 co-regulates cell migration in TNBC cells

Gene ontology analysis of biological process of the commonly AS regulated genes in NEK2- and Sam68 silenced MDA-MB-231 cells compared to control (p-value ≤0.05).
